# I. Diabetes, Mellitus and Insipidus. II. Education and Culture as Relate to the Health and Diseases of Women

**Published:** 1890-03

**Authors:** 


I. **Diabetes, Mellitus and Insipidus.** By ANDREW H. SMITH, M. D., Professor of Clinical Medicine and Therapeutics at the New York Post-Graduate Medical School; Physician to the Presbyterian Hospital, etc., etc. The Physicians' Leisure Library. Detroit: George S. Davis. 1889.

II. **Education and Culture as Relate to the Health and Diseases of Women.** By ALEX. J. C. SKENE, M. D. The Physicians' Leisure Library. Detroit: George S. Davis. 1889.

I. In this brochure of five chapters and seventy-five pages will be found an intelligent setting forth of the latest professional thought on these two forms of diabetes. Four chapters are devoted to the consideration of diabetes mellitus, in which its clinical history and complications; its pathology and morbid anatomy; its causes, diagnosis, and progress; and its prevention and treatment, are carefully considered. The remaining chapter is devoted to the insipid variety of the disease, or polyuria.

II. The great importance of the subject of education and culture as correlated to Health and Diseases of Women, is beginning to be appreciated by physicians, and Dr. Skene has presented an interesting essay upon it that ought to be extensively and carefully read. The late Dr. E. H. Clark, in his Sex in Education, began a work in this field that is bearing good fruit, and this latest contribution to the subject—for these two treatises have an unmistakable kinship—is one every way worthy of both title and author. The price of these two late additions to the Physicians' Leisure Library is the same as the others, viz.: in paper, 25 cents; and in cloth, 50 cents.

